# Evaluation of efflux pump activity and biofilm formation in multidrug resistant clinical isolates of *Pseudomonas aeruginosa* isolated from a Federal Medical Center in Nigeria

**DOI:** 10.1186/s12941-021-00417-y

**Published:** 2021-02-02

**Authors:** Florence Chijindu Ugwuanyi, Abraham Ajayi, David Ajiboye Ojo, Adeyemi Isaac Adeleye, Stella Ifeanyi Smith

**Affiliations:** 1 Department of Microbiology, University of Lagos Akoka, Lagos, Lagos State Nigeria; 2 Molecular Biology and Biotechnology Department, Nigerian Institute of Medical Research (NIMR) Yaba, Lagos, Lagos State Nigeria; 3 Federal University of Agriculture Abeokuta (FUNAAB), Abeokuta, Ogun State Nigeria; 4 Department of Biological Sciences, Mountain Top University, Makogi Oba, Ogun State Nigeria

**Keywords:** Biofilm, Efflux pump, *Pseudomonas aeruginosa*, Antibiotic resistance

## Abstract

**Background:**

*Pseudomonas aeruginosa* an opportunistic pathogen, is widely associated with nosocomial infections and exhibits resistance to multiple classes of antibiotics. The aim of this study was to determine the antibiotic resistance profile, biofilm formation and efflux pump activity of *Pseudomonas* strains isolated from clinical samples in Abeokuta Ogun state Nigeria.

**Methods:**

Fifty suspected *Pseudomonas* isolates were characterized by standard biochemical tests and PCR using *Pseudomonas* species -specific primers. Antibiotic susceptibility testing was done by the disc diffusion method. Efflux pump activity screening was done by the ethidium bromide method and biofilm formation assay by the tissue plate method. Genes encoding biofilm formation (*pslA* & *plsD*) and efflux pump activity (*mexA, mexB* and *oprM*) were assayed by PCR.

**Results:**

Thirty-nine *Pseudomonas* spp. were identified of which 35 were *Pseudomonas aeruginosa* and 4 *Pseudomonas* spp. All 39 (100%) *Pseudomonas* isolates were resistant to ceftazidime, cefuroxime and amoxicillin-clavulanate. Thirty-six (92%), 10(25.6%), 20 (51.2%), 11(28%) and 9(23%) of the isolates were resistant to nitrofurantoin, imipenem, gentamicin, cefepime and aztreonam respectively. All the isolates had the ability to form biofilm and 11 (28%) of them were strong biofilm formers. They all (100%) harboured the *pslA* and *pslD* biofilm encoding genes. Varied relationships between biofilm formation and resistance to ciprofloxacin, ofloxacin, cefixime, gentamicin, imipenem, and aztreonam were observed. Only 23(59%) of the *Pseudomonas* isolates phenotypically exhibited efflux pump activity but *mexA* gene was detected in all 39 (100%) isolates while *mexB* and *oprM* genes were detected in 91%, 92%, and 88% of strong, moderate and weak biofilm formers respectively.

**Conclusion:**

Multidrug resistance, biofilm and efflux pump capabilities in *Pseudomonas aeruginosa* have serious public health implications in the management of infections caused by this organism.

## Background

Treatment options available for mild and severe bacterial infections are now limited due to multidrug resistance making treatment difficult and expensive. Some bacteria possess intrinsic mechanisms that facilitate resistance to multiple antibiotics, while some acquire this capability through horizontal gene transfer. *Pseudomonas* species, most especially the opportunistic pathogen *Pseudomonas aeruginosa* are known to exhibit large intrinsic resistance to multiple antibiotics across most classes including aminoglycoside, fluoroquinolones and β-lactams (third and fourth generation cephalosporins, carbapenem, monobactam). Multidrug resistant (MDR) *P. aeruginosa* strains have been implicated in urinary tract infections, bacteremia, respiratory tract infections and wound infections [1–4]. Two prominent mechanisms, biofilm formation and efflux pump activity among other mechanisms play conspicuous role in persistence and antibiotic resistance of infecting *Pseudomonas* spp. Extruding action of bacteria efflux pumps prevent or limit antimicrobials from reaching their target site within the cell. About twelve resistance-nodulation-division (RND) family of efflux pumps have been identified in *P. aeruginosa* four of which mediate antibiotic resistance [5, 6]. The *mexAB*-*oprM* and *mexXY*-*oprM* multidrug efflux pump systems of *P. aeruginosa* are said to be involved in acquired and intrinsic resistance, while the other two systems only mediate acquired resistance [3]. The *mexAB*-*oprM* efflux pump system in particular have been reported to contribute to intrinsic resistance of *P. aeruginosa* to chloramphenicol, β-lactams, quinolones, macrolides, tetracycline and novobiocin [7]. Beyond resistance, these efflux pumps systems also have an interplay in stress response and virulence [8]. Adhesion of *P. aeruginosa* to host cell via the type IV pili coupled with the secretion of extracellular polysaccharide mediated by polysaccharide synthesis locus (*psl*) facilitates the formation of biofilm which promote antibiotic resistance and shields the pathogen from hosts’ immune system [9]. Biofilm formation by *P. aeruginosa* facilitate recalcitrant and severe clinical outcomes in patients with cystic fibrosis, wound infection and compromised immune system [10, 11]. There have been several reports on antibiotic resistant clinical *P. aeruginosa* isolates and associated resistance genes in Nigeria [12–14]. However there is a draught of information correlating biofilm formation and efflux pump activity with antibiotic resistance profile of *P. aeruginosa.* This present study evaluates efflux pump activity, biofilm forming potential and antibiotic resistance profile of *Pseudomonas* species isolated from clinical samples in Nigeria.

## Methods

### Bacterial strains

Fifty suspected *Pseudomonas* spp. that were isolated from various clinical samples and preserved in agar slants were obtained between April and September 2019 from the Federal Medical Center Abeokuta a tertiary medical health facility in Ogun state, South western Nigeria. Eleven suspected *Pseudomonas* isolates were of urine origin, 3 were from eye swab and 12 each were from wound and ear swabs respectively as shown in Table 1. To characterize *Pseudomonas* strains, isolates were inoculated into Brain Heart Infusion (BHI) (Oxoid, Basingstoke, UK) broth from agar slants and incubated at 37 °C for 18 h. Bacteria broth culture was then streaked onto nutrient agar (Oxoid, Basingstoke, UK) and cetrimide agar (Merck Darmstadt, Germany) and incubated at 37 °C for 24 h. This was followed by standard biochemical tests including Simmon’s citrate, oxidase, catalase, hydrogen sulphide production, sugar fermentation and gas production. Species- specific PCR using the primer pair PA-SSF and PA-SSR shown in Table 2 was used in characterizing *Pseudomonas aeruginosa*.

**Table 1:** Quantity of suspected *Pseudomonas aeruginosa* isolated from various clinical samples

S/N	Clinical sample	Number of suspected *Pseudomonas* isolates
1	Urine	11
2	Eye swab	3
3	Pus	4
4	Sputum	3
5	Wound swab	12
6	Ear swab	12
7	Catheter tip	3
8	Aspirate	1
9	Skin swab	1
Total		50

S/N: Serial number

**Table 2:** Lists primers for species specific (*P. aeruginosa*), efflux pumps and biofilm forming genes used in this study

Primer	Sequence	Product size (bp)	Annealing temperature (^°^C)	Reference
*mexA*-*F* *mexA*-*R*	5′-ACCTACGAGGCCGACTACCAGA-3′ 5′-GTTGGTCACCAGGGCGCCTTC-3′	179	57	[20]
*mexB*-*F* *mexB*-*R*	5′-GTGTTCGGCTCGCAGTACTC-3′ 5′-AACCGTCGGGATTGACCTTG-3	244	56	[20]
*oprM*-*F* *oprM*-*R*	5′-CCATGAGCCGCCAACTGTC-3′ 5′-CCTGGAACGCCGTCTGGAT-3′	205	57	[20]
*pslA*-*F* *pslA*-*R*	5′-TGGGTCTTCAAGTTCCGCTC-3′ 5′-ATGCTGGTCTTGCGGATGAA-3′	119	55	[21]
*pslD*-*F* *pslD*-*R*	5′-CTCATGAAACGCACCCTCCT-3′ 5′-TGCGACCGATGAACGGATAG-3′	295	52	[21]
*PA*-*SSF* *PA*-*SSR*	5′-GGGGATCTTCGGACCTCA-3′ 5′-TCCTTAGAGTGCCCACCCG-3′	956	56	[22]

### Antimicrobial susceptibility testing

Antimicrobial susceptibility testing was done using the Kirby-Bauer disc diffusion method in accordance with the guideline of the European Committee on Antimicrobial Susceptibility Testing [15]. Bacterial isolates were inoculated into BHI broth and incubated for 18 h at 37 °C. Then broth culture was streaked on nutrient agar (Oxoid, Basingstoke, UK) and incubated for 24 h at 37 °C. One to two colonies were emulsified in sterile normal saline to make a suspension that was adjusted to 0.5 McFarland standard and then applied on the surface of Muller-Hinton agar (Oxoid, Basingstoke, UK) using sterile swab sticks. The following antibiotic discs comprising Ceftazidime (30 µg), Cefuroxime (30 µg), Gentamicin (10 µg), Cefixime (30 µg), Ofloxacin (30 µg), Augumentin (30 µg), Nitrofurantoin (30 µg), Ciprofloxacin (30 µg), Cefepime (30 µg), Aztreonam (30 µg) and Imipenem (10 µg) were then placed on the inoculated Muller-Hinton agar. Results were taken after 24 h incubation at 37 °C. *Pseudomonas aeruginosa* ATCC 27853 and *Escherichia coli* ATCC 25922 were used as quality control isolates for the antimicrobial susceptibility testing. Choice of antibiotics selected for this study was inferred from common antibiotics prescribed by physicians and generally consumed by individuals.

### Phenotypic screening of isolates for efflux pump activity and biofilm formation assay

The Ethidium Bromide (EtBr)-agar cartwheel method as described by Anbazhagan et al. [16] was used to determine the efflux pump activity of isolates. Briefly, bacterial cell suspension of approximately 10^6^ cells per mL was streaked on Mueller–Hinton agar plates containing 0 mg/L, 0.5 mg/L, 1 mg/L, 1.5 mg/L, and 2 mg/L concentrations of EtBr and incubated for 24 h at 37 °C. After incubation, the plates were examined under UV transilluminator (Cleaver Scientific Ltd) for fluorescence and isolates that fluoresced at minimum concentration of EtBr were recorded as not possessing active efflux pumps, while those that did not fluoresce possessed active efflux pumps.

Biofilm formation assay was performed using the tissue culture plate (TCP) method described by Mathur et al. [17]. Single colonies of each *Pseudomonas* isolate were inoculated into brain heart infusion (BHI) broth (Oxoid, Basingstoke, UK) supplemented with 2% sucrose and 200 µL of bacterial suspension was loaded into the individual wells of 96-well microtiter plate. Two hundred microlitres of sterile BHI broth was also loaded into a well as negative control. Plates were incubated at 37 °C for 24 h after which contents of each well was discarded and wells were washed three times with sterile deionized water to remove non-adherent bacteria. Wells were air-dried for 45 min and 200 µL of 0.1% (v/v) crystal violet solution was added to each well and incubated for 45 min at room temperature. Thereafter, the wells were again washed four times with sterile deionized water. Incorporated dye was solubilized by adding 200 µL of 33% (v/v) glacial acetic acid to each well and optical density (OD) was measured at 650 nm using the Emax^®^ Plus Microplate Reader (Molecular Devices San Jose, CA). The assay was performed in triplicates and biofilm forming potential were recorded as mean OD values as described by Hassan et al. [18] to be strong (≥ 0.108), moderate (0.108–0.083) and weak (< 0.083).

### Molecular characterization of isolates, efflux pump coding genes and biofilm coding genes

Genomic DNA of *Pseudomonas* isolates was extracted according to the method of Kpoda et al. [19]. Cell suspension was made from 3 to 5 colonies of overnight bacterial culture in 1 mL of sterile water and suspension was held for 10 min at 100 °C in a thermomixer (Eppendorf Hamburg Germany). Suspension was centrifuged at 13,000 rpm for 10 min at 4 °C and supernatant containing DNA was separated and stored at − 20 °C for subsequent use. PCR reaction targeting efflux pump genes (*mexA, mexB* and *oprM*), biofilm formation genes (*pslA* and *pslD*) and *PA*-*SS* gene (*Pseudomonas aeruginosa* specific) was done in a 20 µL reaction targeting specific primers listed in Table 2. A simplex PCR was used for amplification of *mexA, oprM, pslA*, *pslD*, *mexB* and *PA*-*SS*. PCR reaction mix contained 9.8 µL nuclease free water, 4 µL 5X FIREPOL Solis Biodyne (Tartu, Estonia) master mix, 0.6 µL each of both forward and reverse primers and 5 µL of DNA template. Template DNA sample from *Pseudomonas aeruginosa* ATCC 27853 was used as positive control, while nuclease free water was used as negative control. PCR cycling parameter was initial denaturation at 95 °C for 5 min and 30 cycles of denaturation at 95 °C for 30 s, annealing temperature at (Table 2) for 40 s, elongation at 72 °C for 1 min and final elongation at 72 °C for 10 min carried out in a thermal cycler (Eppendorf AG, Hamburg, Germany). PCR products were electrophoresed in 2% agarose gel at 100 V for 60 min and visualized under a trans-illuminator (Cleaver Scientific Ltd) and a Solis Biodyne 100 bp DNA ladder (Tartu, Estonia) was used as a molecular marker.

### Statistical analysis

Statistical analysis and graphics were performed with IBM SPSS Statistics 20 and Microsoft Excel (Microsoft Cooperation, 2013 USA). Spearman rank correlation test was used to determine the association of biofilm formation and antibiotic susceptibility of isolates. Positive Spearman rank correlation coefficient (ρ) value of +1 was considered as perfectly positive correlation and negative ρ value of − 1 was considered as perfectly negative correlation. Also ρ value less than 0.3 or greater than − 0.3 was considered negligible [23].

## Results

### Bacterial strains

**Fig. 1:**
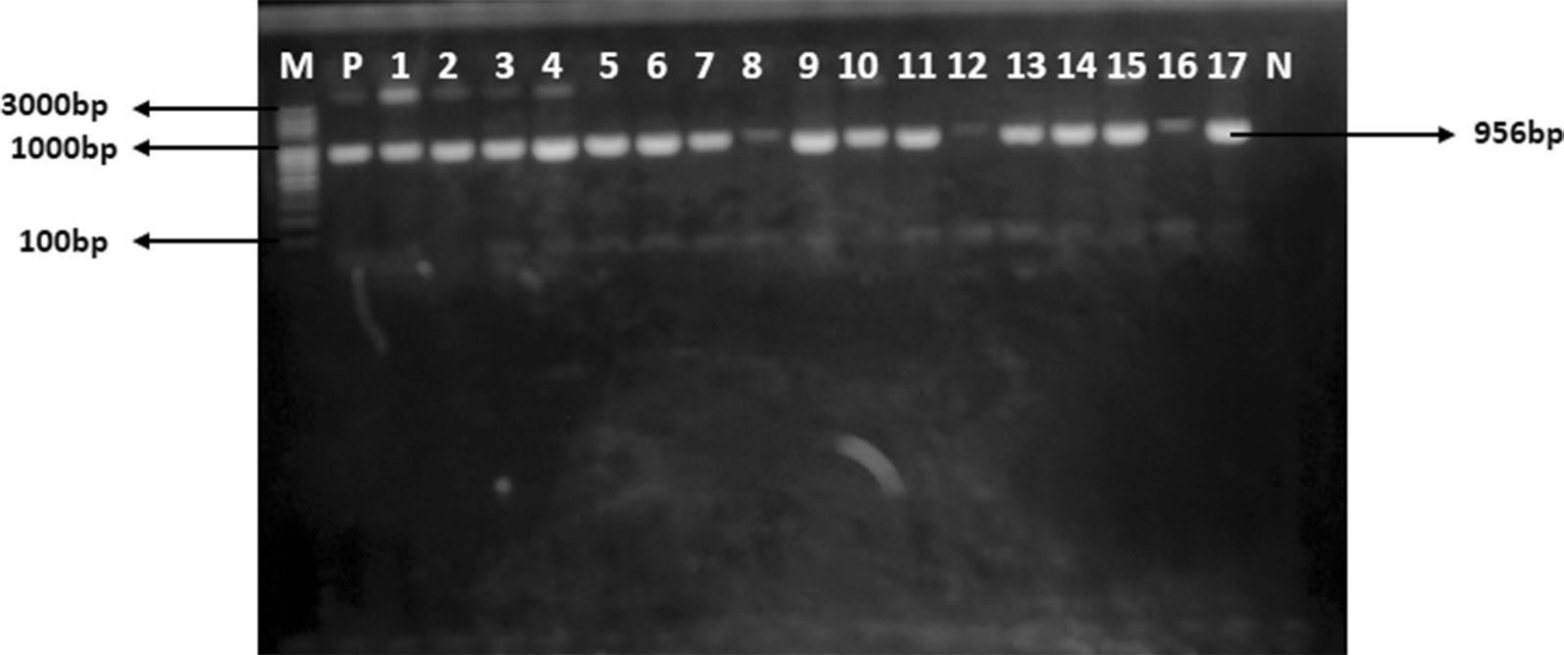
Electrophoretogram showing amplicons of *Pseudomonas aeruginosa* specific primers (*PA*-*SS*). Lane M: 100 bp molecular maker, P: positive control, N: negative control, Lane 1-17 positive for *PA*-*SS*

Of the 50 suspected *Pseudomonas* isolates characterized, 39 were confirmed to be *Pseudomonas* species of which 35 were *Pseudomonas aeruginosa* as they were positive for the *Pseudomonas* species specific gene (*PA*-*SS*) as shown in Fig. 1.

### Antimicrobial Susceptibility

**Fig. 2:**
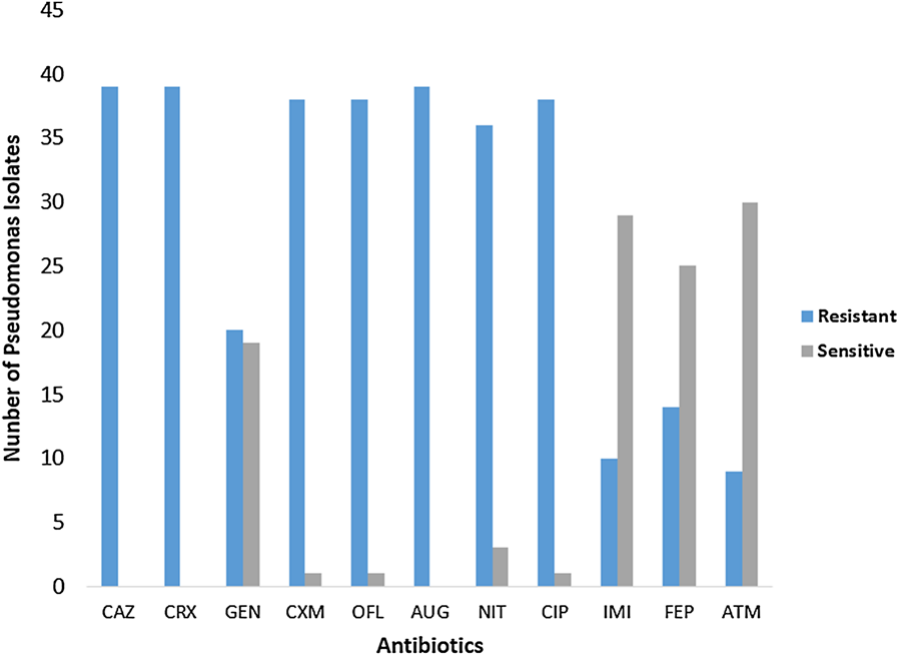
Antibiotic susceptibility profile of *Pseudomonas* isolates. CAZ: Ceftazidime; CRX: Cefuroxime; GEN: Gentamicin; CXM: Cefixime; OFL: Ofloxacin; AUG: Amoxicillin-clavulanate; NIT: Nitrofurantoin; CPR: Ciprofloxacin; IMI: Imipenem; FEP: Cefepime

All the 39 (100%) *Pseudomonas* isolates were resistant to ceftazidime, cefuroxime and amoxicillin-clavulanate. Thirty-eight (97%) isolates were also resistant to cefixime, ofloxacin and ciprofloxacin. Thirty- six (92%) were resistant to nitrofurantoin, 10 (25.6%) were resistant to imipenem, 20 (51.2%) were resistant to gentamicin, 11 (28%) to cefepime and 9 (23%) to aztreonam as shown in Fig. 2.

### Biofilm formation and antibiotic resistance

*Pseudomonas* isolates were categorized into strong, moderate and weak biofilm formers. Eleven (28%), 12 (31%) and 16 (41%) were strong, moderate and weak biofilm formers respectively. Irrespective of the isolates biofilm forming capabilities all isolates (100%) were resistant to ceftazidime, cefuroxime and amoxicillin + clavulanic acid. However, there was a positive correlation between biofilm formation and resistance to ciprofloxacin, ofloxacin and gentamicin as shown in Table 3. However, there was perfectly negative correlation between biofilm formation and resistance to imipenem and aztreonam. While the association between resistance to nitrofurantion and biofilm formation was negligible as correlation coefficient was − 0.25.

**Table 3:** Strong, moderate and weak biofilm formers and their percentage resistance to various antibiotics

Antibiotics	Resistance (%)			Ρ
	SBF (%) n = 11	MBF (%) n = 12	WBF (%) n = 16	
Ceftazidime	100	100	100	a
Cefuroxime	100	100	100	a
Gentamicin	45.5	58.3	50	0.5
Cefixime	100	91.7	100	0.5
Ofloxacin	100	100	93.8	0.5
Amoxicillin + clavulanic acid	100	100	100	a
Nitrofurantoin	90.9	91.7	93.8	− 0.25
Ciprofloxacin	100	91.7	100	0.5
Imipenem	45.5	25	12.5	− 1
Cefepime	9.1	50	25	− 0.5
Aztreonam	45.5	25	6.25	− 1

SBF: Strong biofilm formers, MBF: Moderate biofilm formers, WBF: Weak biofilm formers, ρ: Spearman rank correlation coefficient, a: ρ was not determined

All (100%) Strong biofilm formers (SBF) and moderate biofilm formers (MBF) were resistant to ofloxacin while 93.8% of weak biofilm formers (WBF) were resistant to the same antibiotic. Both SBF and WBF were 100% resistant to ciprofloxacin while 91.7% of MBF were resistant to ciprofloxacin. SBF displayed low level of resistance (9.1%) to cefepime, while 50% of both MBF and WBF were resistant to cefepime.

### Efflux pump evaluation and biofilm capability

**Fig. 3:**
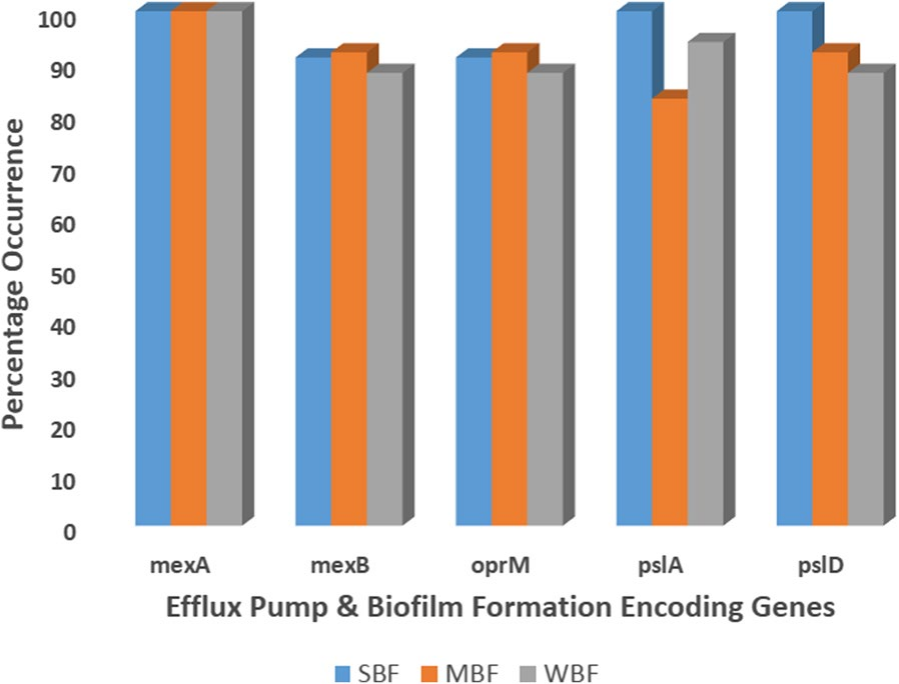
Percentage occurrence of efflux pump and biofilm formation encoding genes. SBF: Strong biofilm formers, MBF: Moderate biofilm formers, WBF: Weak biofilm formers

At 1 mg/L and 1.5 mg/L of EtBr, distinct efflux pump activity was observed as 23 (59%) of *Pseudomonas* isolates phenotypically exhibited efflux pump activity while 16(41%) did not. However, all (100%) *Pseudomonas* isolates possessed *mexA* gene a multidrug efflux pump gene that belong to the resistance-nodulation-division–division (RND) family. *mexB* and *oprM* genes members of the RND family as mexA were also detected in 91%, 92%, and 88% of strong, moderate and weak biofilm forming *Pseudomonas* isolates respectively as shown in Fig. 3 and all 23 *Pseudomonas* isolates that phenotypically exhibited efflux pump activity possessed both genes. Beyond phenotypic exhibition of biofilm formation, genotypic evaluation revealed that all SBF possessed the *pslA* and *pslD* genes while 83% and 92% of MBF harboured *pslA* and *pslD* respectively. Ninety-four percent of WBF possessed *pslA* and 88% had *pslD* genes.

## Discussion

To our knowledge this study is the first in which the biofilm forming potential and efflux pump activity of multidrug resistant *Pseudomonas* species isolated from clinical samples were evaluated in Nigeria. Thirty- five (89.7%) of the 39 phenotypically identified *Pseudomonas* isolates were *Pseudomonas aeruginosa* indicating that it is wide spread in nosocomial infections as previously reported [24]. Managing infections caused by *Pseudomonas* strains are often challenging since they exhibit resistance to most classes of antibiotics [25]. In this study, all (100%) *Pseudomonas* isolates were resistant to ceftazidime, cefuroxime and amoxicillin-clavulanate, while 38 (97%) were resistant to cefixime, ofloxacin, ciprofloxacin and 36 (92%) were resistant to nitrofurantoin. Odumosu et al. [26] in their study in southwestern Nigeria also reported a 100% resistance of *Pseudomonas* isolates to amoxicillin-clavulanate, 87.1% resistance to ceftriaxone but a lower percentage (22.5) resistance to ceftazidime. The emergence of carbapenem and monobactam resistant *Pseudomonas* species have been reported by several workers [27–29]. Similarly, 9 (23%), 11 (28%) and 10 (25.6%) of *Pseudomonas* isolates in this study were resistant to aztreonam, cefepime and imipenem respectively.

Biofilm formation enhances antibiotic resistance and virulence in *Pseudomonas aeruginosa* resulting in persistence of lingering infections [30]. In this study all *Pseudomonas* isolates had the ability to form biofilm. However, 11 (28%) of them were strong biofilm former (SBF), while 12 (31%) and 16 (41%) were moderate (MBF) and weak (WBF) biofilm formers respectively. Furthermore, there was a positive correlation between observed resistance to ofloxacin, ciprofloxacin and gentamicin and biofilm formation. Although correlation factor for ofloxacin and ciprofloxacin were positive it would be difficult to ascertain the role of biofilm formation in the observed antibiotic resistance as majority (38) of the *Pseudomonas* isolates were resistant to both antibiotics. However, Cepas et al. [31] have reported a correlation between ciprofloxacin resistance and biofilm formation in *P. aeruginosa*. Similarly, Mohammed and Abd Alla [32] in Egypt reported the resistance of biofilm forming *P. aeruginosa* isolates to ciprofloxacin, tobramycin and gentamycin compared to their non-biofilm forming counterpart that were susceptible. In contrast we observed a positive relationship between resistance to gentamicin and biofilm formation. It has also been reported in *P. aeruginosa* that resistance to imipenem results in reduced biofilm formation [33]. This might explain the perfectly negative correlation between biofilm formation and resistance to imipenem observed in this study. A similar association was also observed with the antibiotic aztreonam. Adhesion and secretion of extracellular polysaccharide are requisites to initiate biofilm formation in bacteria. The *pslA* and *pslD* genes which are key components of the polysaccharide synthesis locus (*psl*) responsible for the secretion of extracellular polysaccharide in *Pseudomonas aeruginosa* were detected in majority of our *Pseudomonas* isolates. All (100%) SBF harboured both *pslA* and *pslD* genes while MBF and WBF possessed these genes in various proportions. In a similar study in Yaoundé Cameroon, Madaha et al. [34] reported the detection of *pslA* gene in 92% of antibiotic resistant *Pseudomonas aeruginosa* clinical isolates.

Apart from biofilm formation that contributes to antibiotic resistance, active efflux pump systems are prominent mechanisms that also mediate multidrug resistance in bacteria. The *mexAB*-*OprM* efflux pump identified to have a high level of expression in *P. aeruginosa* concomitantly driving resistance to multiple classes of antibiotics [35] was detected in this study. *mexA* gene was detected in all (100%) *Pseudomonas* isolates. This is similar to the report of Abbas et al. [24] who reported the detection of *mexAB*-*R* in all *P*. *aeruginosa* isolated from urinary tract infections in Egypt. Although the direct role of efflux pump in biofilm formation was not evaluated in this study, we observed that 91%, 92% and 88% strong, moderate and weak biofilm formers respectively were positive for *mexB* and *oprM* genes. Possession of these genes could have an interplay in the virulence of the pathogen. Alav et al. [36] in their study indicated the role of these efflux pump genes in biofilm formation in *P*. *aeruginosa* apart from extrusion of drug. Thus efflux pumps may be playing a dual role in this organism.

## Conclusion

*Pseudomonas aeruginosa* isolates in this study were resistant to various antibiotics and had biofilm forming potential. The strains showed varied relationship between biofilm formation and resistance to some of the test antibiotics. These findings emphasize the health risks and difficulty that could be encountered in eradicating *P*. *aeruginosa* infections. Therefore scrupulous hygiene practice and antibiotic surveillance are important to prevent continuous spread of infection and mortality caused by this organism.

## Data Availability

Data sets used and analysed for this study are available from the corresponding author on reasonable request. All data generated or analysed during this study are also included in this published article.

## Abbreviations

EtBr: Ethidium Bromide
EUCAST: European Committee on Antimicrobial Susceptibility Testing
RND: Resistance-nodulation-division
MDR: Multidrug resistance
OD: Optical density
SBF: Strong biofilm formers
MBF: Moderate biofilm formers
WBF: Weak biofilm formers
